# Neural stem cells genetically-modified to express neprilysin reduce pathology in Alzheimer transgenic models

**DOI:** 10.1186/scrt440

**Published:** 2014-04-16

**Authors:** Mathew Blurton-Jones, Brian Spencer, Sara Michael, Nicholas A Castello, Andranik A Agazaryan, Joy L Davis, Franz-Josef Müller, Jeanne F Loring, Eliezer Masliah, Frank M LaFerla

**Affiliations:** 1 Department of Neurobiology and Behavior and Institute for Memory Impairment and Neurological Disorders, University of California Irvine, Irvine, CA 92697, USA; 2 Center for Regenerative Medicine, the Scripps Research Institute, La Jolla, CA 92037, USA; 3 Center for Psychiatry (ZIP Kiel), University Hospital Schleswig Holstein, Kiel 24105, Germany; 4 Department of Neurosciences, University of California San Diego, La Jolla, CA 92093, USA

## Abstract

**Introduction:**

Short-term neural stem cell (NSC) transplantation improves cognition in Alzheimer’s disease (AD) transgenic mice by enhancing endogenous synaptic connectivity. However, this approach has no effect on the underlying beta-amyloid (Aβ) and neurofibrillary tangle pathology. Long term efficacy of cell based approaches may therefore require combinatorial approaches.

**Methods:**

To begin to examine this question we genetically-modified NSCs to stably express and secrete the Aβ-degrading enzyme, neprilysin (sNEP). Next, we studied the effects of sNEP expression *in vitro* by quantifying Aβ-degrading activity, NSC multipotency markers, and Aβ-induced toxicity. To determine whether sNEP-expressing NSCs can also modulate AD-pathogenesis *in vivo*, control-modified and sNEP-NSCs were transplanted unilaterally into the hippocampus of two independent and well characterized transgenic models of AD: 3xTg-AD and Thy1-APP mice. After three months, stem cell engraftment, neprilysin expression, and AD pathology were examined.

**Results:**

Our findings reveal that stem cell-mediated delivery of NEP provides marked and significant reductions in Aβ pathology and increases synaptic density in both 3xTg-AD and Thy1-APP transgenic mice. Remarkably, Aβ plaque loads are reduced not only in the hippocampus and subiculum adjacent to engrafted NSCs, but also within the amygdala and medial septum, areas that receive afferent projections from the engrafted region.

**Conclusions:**

Taken together, our data suggest that genetically-modified NSCs could provide a powerful combinatorial approach to not only enhance synaptic plasticity but to also target and modify underlying Alzheimer’s disease pathology.

## Introduction

Alzheimer’s disease (AD) is the leading cause of age-related dementia, afflicting one in every eight people over the age of 65 [1]. Unfortunately, the incidence of dementia is expected to quadruple over the next 40 years, such that more than 115 million people will be afflicted world-wide by 2050 [2]. Currently approved therapies offer only marginal, temporary relief and fail to modify the underlying disease pathology. Thus, there is a critical and urgent need to examine novel and combinatorial approaches to treat this devastating disorder.

Pathologically, AD is characterized by the accumulation of two hallmark brain lesions, beta-amyloid (Aβ) plaques and neurofibrillary tangles (NFTs). Aβ plaques result from the extracellular accumulation of insoluble aggregates of a small 40 to 42 amino acid peptide, Aβ. NFTs in contrast, consist of intraneuronal insoluble aggregates of the microtubule binding protein, tau, which becomes hyperphosphorylated and compromises neuronal activity, connectivity and function. In addition to the accumulation of Aβ and tau across multiple brain regions, AD patients also exhibit inflammation and widespread synaptic and neuronal loss.

Given the complex nature of this disease and the multiple pathways and regions affected, a single small molecule approach may not provide substantial benefit in the absence of other interventions. Biologic-based approaches, such as stem cell transplantation, are therefore receiving increasing attention. Recently, we and others showed that neural stem cell (NSC) transplantation markedly improves cognitive function, synaptic connectivity and neuronal survival in models of AD and tauopathy [3,4]. Many of these effects appear to be mediated by stem cell-derived neurotrophins or other neuroprotective activities. Mesenchymal stem cells have also been found to improve cognition in AD models by modulating cytokine levels and brain inflammation [5]. Thus, stem cells provide therapeutic efficacy in preclinical models of AD by modulating complex biological systems via multiple mechanisms. Although short-term benefits of stem cell transplantation appear promising and warrant further examination, these studies have also shown that NSCs do not modify the underlying Aβ or tau pathology [3,4]. Hence, it is critical to examine combinatorial approaches aimed at not only improving synaptic connectivity and neuronal function but also diminishing Aβ and tau accumulation.

Over the last decade, pathways that underlie the endogenous degradation of Aβ have been identified. Among these, the proteolytic enzyme neprilysin (NEP) exhibits some of the most potent activity [6,7]. Interestingly, levels and activity of NEP are decreased in AD brains, suggesting that a reduction in Aβ degradation may contribute to the development of the disease [8,9]. Approaches that enhance or harness these degradation pathways may provide important disease-modifying efficacy. In support of this, transgenic or viral-mediated overexpression of NEP reduces Aβ pathology [7,10]. Unfortunately, widespread delivery of these therapeutic proteins to the large human brain will likely be required, but is hindered by the blood brain barrier and the limited radius of infectivity (<0.5 mm) afforded by current viral-based approaches [11]. One possible solution to this problem is to harness the migratory activity of NSCs [12] and use these cells to deliver Aβ-degrading enzymes throughout the forebrain.

In this study, we hypothesized that NSCs could provide an effective means to deliver disease-modifying therapeutic proteins to the brain. To test this hypothesis, we generated murine NSC lines that overexpress a secreted form of the Aβ-degrading enzyme, neprilysin (sNEP). *In vitro* characterization of these cells revealed high levels of sNEP protein expression and no changes to NSCs markers or differentiation potential. sNEP expression also protected NSCs against Aβ-induced toxicity both *in vitro* and *in vivo*. Most critically, these cells survive and continue to produce sNEP for several months following transplantation, markedly reducing Aβ pathology and enhancing synaptic connectivity in two independent transgenic models. Thus, sNEP-expressing NSCs represent a promising therapeutic approach that combines the neurotrophic-mediated benefits of stem cell transplantation with the widespread delivery of a disease-modifying protein.

## Methods

### Mice

All animal experiments were approved by the University of California, Irvine Institutional Animal Care & Use Committee and were performed in strict accordance with National Institutes of Health guidelines. The 3xTg-AD and Thy1-APP mice have previously been described [13-15]. 3xTg-AD mice were generated by co-microinjecting transgenes expressing human APP_695_ with the Swedish mutation (KM670/671NL) and human tau with the P301L mutation under control of the murine Thy1.2 promoter into single-cell embryos of homozygous PS1_M146V_ knockin mice. Thy1-APP mice were generated by pronuclear injection of a transgene expressing APP_751_ with Swedish and London (V717I) mutations under control of the murine Thy1 promoter. The 3xTg-AD mice are maintained on a hybrid C57BL6/129 background and Thy1-APP mice are maintained as purebred C57/Bl6. Equivalent numbers of both male and female mice were utilized in this study and housed on a 12-hour light/dark schedule with *ad libitum* access to food and water. Importantly, both C57Bl6 and 129 background strains have the identical major histocompatibility complex (MHC) haplotype (H-2b) as the C57Bl6-derived NSCs utilized in this study.

### Neural stem cells

The neural stem cells used for transplantation into 3xTg-AD mice were isolated from postnatal day 1 GFP-expressing transgenic mice as previously described [16]. NSCs transplanted into Thy1-APP mice were derived from the C57Bl6 E15 cortex (Millipore SCR029). NSC lines were grown as adherent monolayers using standard NSC media ((D)MEM/F12 with Glutamax, N2, epidermal growth factor (EGF)). To generate lines that stably expressed s-NEP or empty vector control (pBOBI), GFP-NSC cultures were transfected with 5 μg of plasmid using an AMAXA nucleofector (program A-33) following standard protocols (Lonza Inc. Alpharetta, GA, USA). Cells were maintained using standard media and three days after nucleofection, selective antibiotic (Zeocin 100 ng/ml, Invivogen, San Diego, California, USA) was added. Media were changed every two days and cells were split as needed. After six weeks of stable selection, cells were frozen down or utilized for experiments. For Thy1-APP studies, NSCs were transduced with lentiviral particles (multiplicity of infection (MOI) = 50) generated by transfection of HEK293 cells with s-NEP or p-BOBI lentiviral and packaging plasmids as described [10]. For transplantation, control and s-NEP-expressing NSCs were lightly trypsinized, washed three times, triturated and filtered through a 70 μm mesh. Cells were then counted and resuspended in vehicle (1× Hanks balanced salt solution with 20 ng/ml hEGF) at a density of 50,000 cells/μl.

### Stereotactic surgeries

Control- and sNEP-transfected NSCs were delivered to the hippocampus or subiculum using a stereotaxic apparatus. For 3xTg-AD mice, NSCs were injected into the subiculum using the following coordinates relative to Bregma: AP: -3.6, ML: ±2.1, DV: -2.0. Thy1-APP mice received intrahippocampal injections using coordinates: AP: -2.0, ML: ±1.5, DV: -1.3. The 3xTg-AD mice were anesthetized with isofluorane, placed in the stereotax and injected with 100,000 control-transfected NSCs into one subiculum/hippocampus (2 μl/injection) and 100,000 sNEP-NSCs into the other hippocampus using a 5 μl Hamilton microsyringe (30-gauge) and an injection rate of 0.5 μl/minute. The side receiving sNEP cells was randomly chosen and recorded for each animal. Thy1-APP mice underwent similar surgeries except that one side of the hippocampus received a vehicle injection whereas the other side received either sNEP- or control-NSCs. Accurate placement of the injection to the targeted region was confirmed for all animals by visualization of the needle tract within coronal brain sections.

### Tissue processing

The 3xTg-AD mice were sacrificed three months after transplantation and the Thy1-APP mice were sacrificed one month after transplantation via Nembutal overdose and cardiac perfusion with 0.01 M phosphate-buffered saline (PBS) followed by 4% paraformaldehyde (pH 7.4). Brains were rapidly removed and post-fixed for an additional 24 hours at 4°C. Prior to sectioning, a notch was made in the bottom left cortex so that left versus right sides could be readily distinguished after sectioning. Brains were then cut in 50 μm thick coronal sections on a Vibratome and stored in PBS with 0.02% NaN_3_ at 4°C until use.

### Immunofluorescent labeling

Fluorescent labelling followed standard protocols as previously described [3,17]. Primary antibodies utilized include: antibodies against Aβ: 6E10 (Signet, Dedham, MA, USA), fibrillar Aβ (OC, gift of C. Glabe), Tau: HT7 (Innogenetics, Alpharetta, GA, USA), phospho-Tau (Ser 199/202, AT8 epitope; Invitrogen, Carlsbad, CA, USA), glial fibrillary acidic protein (GFAP) (Dako, Carpinteria, CA, USA), β-III-tubulin, GFP, GalC, active caspase-3 and CNPase (Millipore, Carlsbad, CA, USA), Doublecortin (Santa Cruz Biotech, Santa Cruz, CA, USA), and synaptophysin (Sigma, St. Louis, MO, USA). Primary antibodies were applied overnight at 4°C and detected with appropriate Alexa Fluor conjugated secondary antibodies (Invitrogen). Specificity of all primary antibodies was confirmed by Western blot and by omission of primary antibody in immunofluorescent labelling (data not shown).

### Biochemical examination

SDS-PAGE western blots and Aβ sandwich ELISAs were performed following standard protocols as previously described [3]. Primary antibodies used for western blot analysis include: nestin, sox-2, musashi-1 (Millipore), NEP/CD10 (Vector Biolabs, Burlingame, CA, USA), and brain-derived neurotrophic factor (BDNF, Santa Cruz Biotechnology). To test the effects of Aβ treatment on NSC viability cells were treated with 10 nM Aβ42 for 24 hours and then lactate dehydrogenase (LDH) release into the media was measured using the CytoTox 96 LDH assay (Promega Corporation, Madison, WI, USA) following the manufacturer’s protocol.

### Confocal microscopy and quantification

Immunofluorescent sections were visualized using a Leica TCS SPE or Biorad 1024 Confocal microscope. To avoid non-specific bleed-through, each laser line was excited and detected independently. All images represent either single confocal Z-slices or Z-stacks. For quantitative analysis, slides were coded and then images captured by a blinded observer using identical laser and detection settings. Grayscale Z-stack images were then analyzed using Image J software. Three square regions of interest (ROIs) were randomly defined within the area to be quantified (for example, subiculum) and then mean pixel intensity was computed by the software for each ROI. The three fields were averaged for each sample and means were compared by paired t-test.

### Statistical analysis

Comparisons between multiple groups were performed using analysis of variance (ANOVA) followed by Fischer’s PLSD *post hoc* tests. Groups were considered significantly different when *P* <0.05 for both the ANOVA and *post hoc* comparisons. Aβ measurements were quantified from opposing sides of the brain from the same individual animals; the data were therefore compared by paired t-test. All statistical analysis was performed using Graphpad Prism software.

## Results

### sNEP-expressing neural stem cells remain multipotent, degrade Aβ *in vitro*, and are resistant to Aβ toxicity

**Figure 1:**
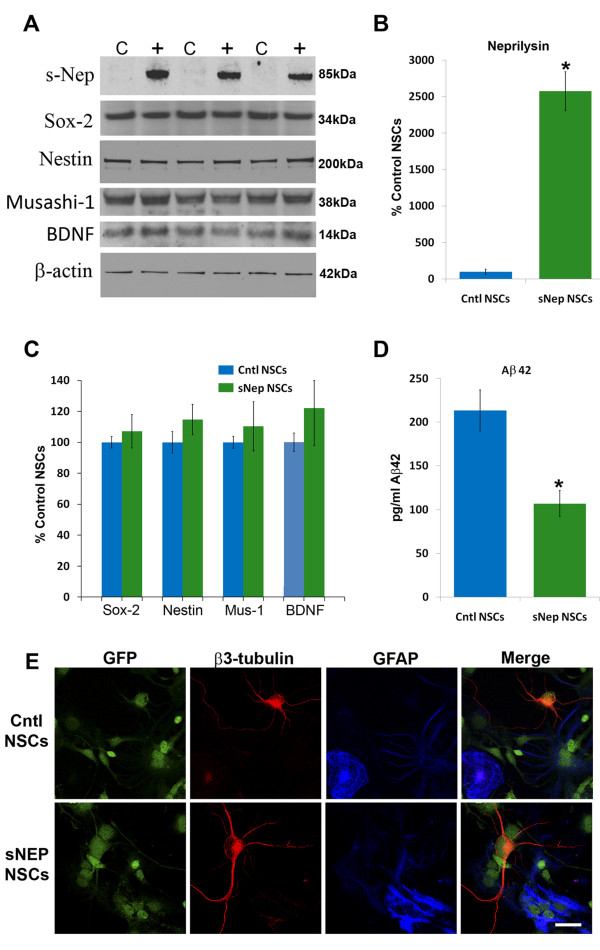
**Stably-transfected sNEP-NSCs remain multipotent and efficiently degrade Aβ. (A)** Western blot analysis of control (C) and sNEP (+) stably-transfected NSCs (+) reveal high levels of sNEP expression versus control transfected NSCs (quantified in **B**). Notably, sNEP expression has no effect on markers of NSC multipotency (sox-2, nestin and musashi-1) and does not alter NSC BDNF expression (quantified in **C**). To determine whether sNEP-expressing cells can degrade Αβ, cultures were treated with recombinant human Aβ42 peptide and 48 hours later, conditioned media were collected and Aβ levels measured by sandwich ELISA. **(D)** sNEP-expressing NSCs efficiently reduced Aβ levels within conditioned media by more than 50% (*P* = 0.0008). **(E)** When NSCs are cultured without mitogen, both control and sNEP-expressing NSCs differentiate into neurons (red, β3-tubulin) and glia (blue, GFAP). N = 10 wells/group, error bars represent standard error of the mean (SEM). Scale Bar = 20 μm. Aβ, beta**-**amyloid; BDNF, brain-derived neurotrophic factor; GFAP, glial fibrillary acidic protein; NSCs, neural stem cells; sNEP, secreted neprilysin.

**Figure 2:**
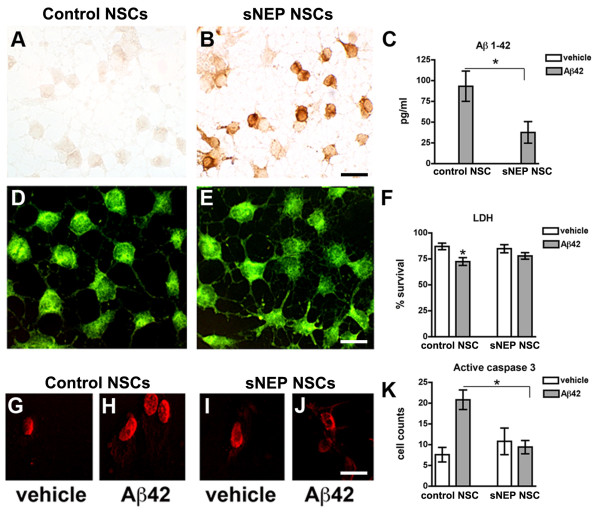
**Independently-generated sNEP-NSCs degrade Aβ and resist Aβ-induced toxicity. (A-B)** Immunohistochemcial labelling confirms high levels of neprilysin expression within lentiviral-transduced NSCs and a significant reduction in Aβ levels (*P* = 0.03) following treatment with recombinant protein **(C)**. No changes are observed in nestin expression **(D-E)**. However, lactate dehydrogenase quantification reveals that sNEP-NSCs are significantly protected from Aβ-induced toxicity **(F)**. Furthermore, activation of caspase-3, an indication of apoptosis, is enhanced following Aβ treatment of control NSCs **(G-H)** but unaltered following treatment of sNEP-NSCs as detected by an anti-active caspase-3 neoepitope antibody **(I-J)**, quantified in **(K)**. N = 3 wells/group. Error bars represent standard error of the mean (SEM). Scale Bar = 30 μm in A-B, 15 μm in D-E, 25 μm in G-J. Aβ, beta**-**amyloid; NSCs, neural stem cells; sNEP, secreted neprilysin.

To determine whether NSCs can be genetically modified to promote Aβ degradation, we stably transfected murine GFP-NSCs with a plasmid encoding sNEP or an empty vector as a control. In parallel, we generated a second line of sNEP-expressing NSCs via lentiviral transduction. sNEP expression and multipotency markers were compared by western blot and immunocytochemistry. As shown, sNEP-expressing NSCs produce over 25 fold higher levels of NEP than control transfected NSCs, which express only low levels of full-length NEP (Figure 1A, Figure 2A,B). To determine whether sNEP expression in any way alters NSC multipotency, we examined three well-established NSC markers, sox-2, nestin, and musashi-1. Western blot analysis confirmed that sNEP- and control-transfected NSCs express equivalent levels of these multipotency markers (Figure 1A,C). Likewise, immunocytochemistry confirmed equivalent expression of the intermediate filament protein, nestin within virally-transduced sNEP and control NSCs (Figure 2D,E). We previously showed that NSCs can improve cognition in AD transgenic mice by a mechanism involving BDNF [3]. Hence, we examined BDNF expression by western blot and verified that levels of this important protein were also unchanged (Figure 1A,C). To further confirm that sNEP-expressing NSCs remain multipotent, cells were allowed to differentiate spontaneously by removing EGF from the culture medium and growing cells in neurobasal media supplemented with B27. One week later, cultures were examined by immunofluoresence, confirming equivalent neural and glial differentiation of sNEP- and control-transfected NSCs (Figure 1E).

To determine whether sNEP-expressing NSCs can efficiently degrade Aβ, cultures of control- and sNEP-expressing NSCs were treated with recombinant human Aβ42 peptide and, 48 hours later, Aβ levels remaining within the media were measured by ELISA. Both the stably-transfected and lentiviral-transduced NSC lines efficiently degraded Aβ *in vitro*, reducing Aβ levels by more than 50% (Figure 1D, *P* = 0.0008 and Figure 2C, *P* = 0.03). Interestingly, we found that treatment of NSCs with 10 nM Aβ42 can trigger caspase-mediated cell death of NSCs within 24 hours (Figure 2). However, sNEP-expression significantly protects NSCs as evidenced by decreased LDH release and reduced caspase-3 activation versus Aβ-treated control NSCs (*P* = 0.03).

### Transplanted sNEP-NSCs secrete neprilysin and degrade Aβ *in vivo*

Having verified that sNEP expression can promote Aβ degradation *in vitro*, we next performed stereotactic surgery to deliver sNEP-NSCs and control-NSCs into the brains of transgenic AD mice. NSCs were targeted to the subiculum or hippocampus of AD transgenic mice as both these regions develop robust Aβ plaque pathology, exhibit significant synaptic degeneration and are critical for learning and memory. Importantly, the hippocampus and subiculum are also dramatically affected in AD patients. Two complementary paradigms were pursued to deliver NSCs. In one set of experiments, 18-month-old 3xTg-AD transgenic mice were transplanted unilaterally with 100,000 sNEP-expressing NSCs into the subiculum and 100,000 control-transfected NSCs into the contralateral subiculum. Three months later, the 3xTg-AD mice were sacrificed and the brains examined. In the second paradigm, 9- month-old Thy1-APP transgenic mice were transplanted with 100,000 sNEP or control-transduced NSCs unilaterally into the hippocampus. The contralateral hippocampus received an equivalent injection of vehicle. One month after transplantation Thy1-APP mice were sacrificed and the brains examined.

**Figure 3:**
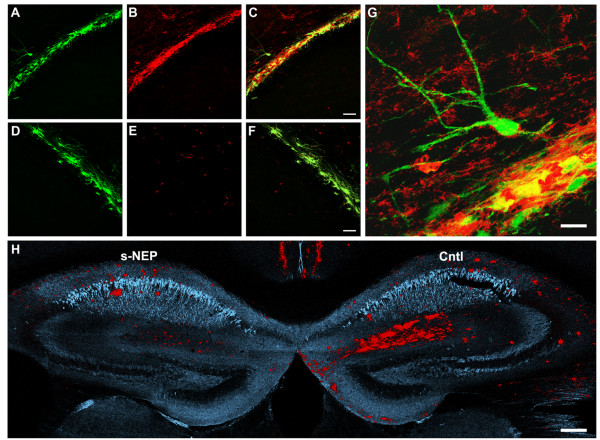
**Genetically-modified NSCs continue to produce neprilysin three months after unilateral transplantation and decrease Aβ load within the ipsilateral hippocampus of aged 3xTgAD mice. (A-C)** Confocal microscopy reveals transplanted NSCs (green, GFP) within the alveus of the hippocampus (white matter tract overlying CA1) that secrete high levels of neprilysin (red) three months post-transplantation. **(D-F)** In contrast, control-transfected NSCs (green, GFP) show no detectable expression of neprilysin (red) *in vivo*. **(G)** High magnification of a sNEP-NSC-derived neuron shows high levels of sNEP (red) surrounding the engrafted cells. **(H)** Three months after transplantation, Aβ plaques (red, OC antibody) within the left side of the hippocampus (sNEP) are significantly reduced versus the contralateral hippocampus (Cntl). No obvious reduction in tau (blue, HT-7 antibody) is observed in aged animals, in line with prior findings that well-established insoluble NFTs are not decreased by Aβ-immunotherapy [19]. Scale Bar = 45 μm in A-F, 15 μm in G, and 160 μm in H. Aβ, beta**-**amyloid; NFTs, neurofibrillary tangles; NSCs, neural stem cells; sNEP, secreted neprilysin.

As previously shown [3], GFP-NSCs engraft well into the hippocampus and migrate into the surrounding white matter tracts and overlying cortex (Figures 3A-F and 5G-L). Notably, sNEP-expressing NSCs continue to produce and secrete NEP into the surrounding parenchema for up to three months post-transplantation (Figure 3A-C). In contrast, NEP expression is not detected adjacent to control-transfected NSCs (Figure 3D-F). Confocal imaging reveals NEP surrounding GFP-expressing cell transplants and also diffusing into the adjacent host parenchyma (Figure 3G).

**Figure 4:**
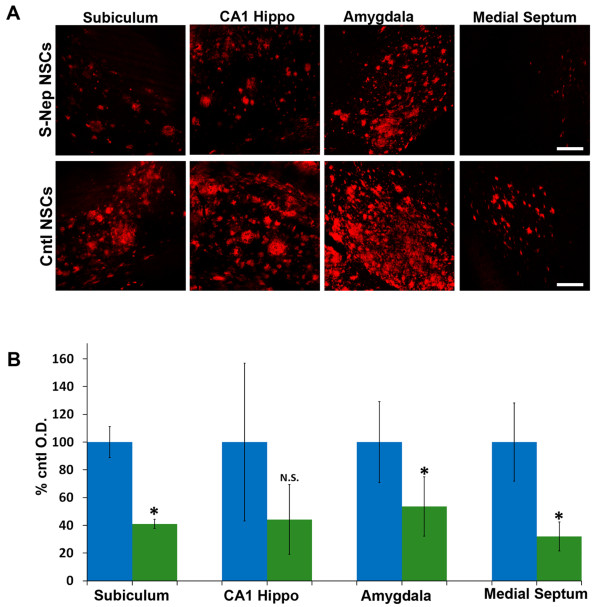
**sNEP-expressing NSCs decrease Aβ plaque load within the subiculum and its efferent targets. (A)** Three months post transplantation, Aβ plaque load (red, 6E10) is reduced by as much as 60% within several regions ipsilateral to the sNEP-expressing NSC grafts. As quantified in **(B)**, Aβ plaques are significantly reduced within the subiculum (*P* = 0.007) directly adjacent to the transplant site, whereas reductions within CA1 fail to reach significance (*P* = 0.18). Interestingly, Aβ load is also significantly reduced within the amygdala (*P* = 0.039) and medial septum (*P* = 0.05), two regions that receive inputs directly from the subiculum. Aβ measurements were quantified from opposing sides of the brain from the same individual animals (n = 5); the data were, therefore, compared by paired t-test. Error bars represent standard error of the mean (SEM). Scale Bar = 140 μm. Aβ, beta**-**amyloid; NSCs, neural stem cells; sNEP, secreted neprilysin.

More importantly, when Aβ levels were assessed, we observed significant reductions in plaque density in areas adjacent to sNEP-NSC grafts (Figure 3H). Interestingly, reductions in Aβ plaque load occurred not only directly adjacent to subiculum grafts, but also within the amygdala and medial septum, two regions that receive substantial inputs directly from the subiculum (Figure 4) [18]. Thus, it appears that sNEP expression may modulate production or transport of Aβ within efferent axonal projections. Plaque load was also examined in the cingulate and piriform cortices. Although NEP expression tended to reduce Aβ in these two regions, these differences failed to reach significance (*P* = 0.27 and 0.32, data not shown). A likely explanation for this finding is that cortical plaque load between individual mice varies more than other quantified regions.

### Established tangle pathology is unchanged but synaptic density is increased by sNEP-NSCs

**Figure 5:**
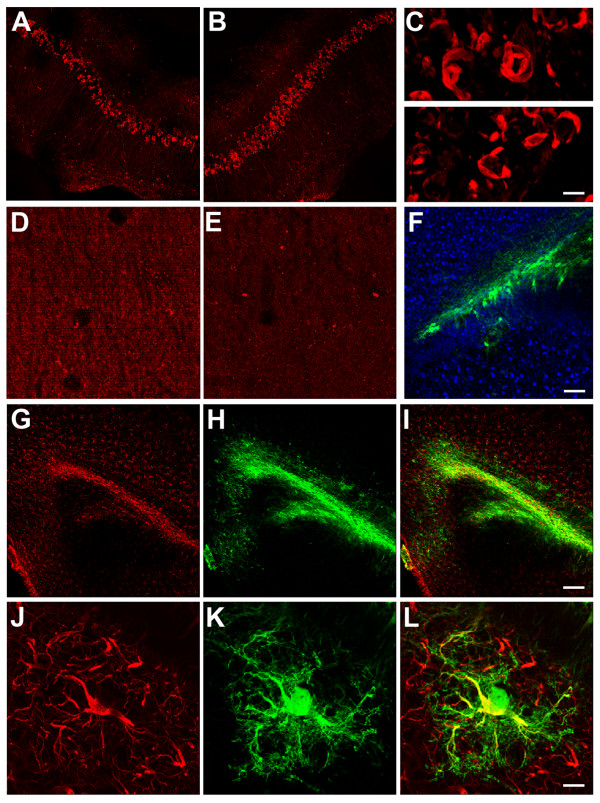
**sNEP- NSCs do not alter established tangle pathology but increase synaptic density and differentiate primarily into astrocytes.** Aged 3xTg-AD mice develop both Aβ plaques and neurofibrillary tangles. We, therefore, examined whether sNEP-NSCs modulate tau pathology *in vivo*. Immunofluorescent labelling and quantification of phosphorylated tau (AT8 epitope: ser199/202) within the hippocampus revealed no significant differences between sNEP-NSC **(A, C-top panel)** and control-NSC **(B, C-bottom panel)** treated sides (n = 5). This finding is in line with previous reports that decreasing Aβ in aged 3xTg-AD mice does not reduce established insoluble tangle pathology. To determine whether sNEP expression leads to a functional effect on neuronal connectivity, we examined synaptophysin immunoreactivity. As shown, sNEP-NSCs increase synaptic density within the subiculum **(D)** by more than 31% versus control-NSCs (**E**, *P* = 0.009, paired t-test). To examine the differentiation of transplanted NSCs, double-labelling for neuronal and glial markers was performed. In agreement with previous studies, very few NSCs (green) transplanted into the aged brain co-express the neuronal marker NeuN (blue, **F**). In contrast, the great majority of NSCs co-express the astrocytic marker GFAP (red, **G-L**). Scale Bar = 100 μm in A-B, 15 μm in C, 6 μm in D-E, 15 μm in F, 300 μm in G-I, and 15 μm in J-L. Aβ, beta**-**amyloid; GFAP, glial fibrillary acidic protein; NSCs, neural stem cells; sNEP, secreted neprilysin.

Previous studies have shown that therapies that decrease Aβ can also reduce tau accumulation and hyperphosphorylation in young 3xTg-AD mice [19]. However, in aged transgenic mice with well-established NFT pathology, Aβ-targeted therapies fail to modify tau. To determine whether sNEP-NSCs can alter tau pathology in aged 3xTg-AD mice we quantified the levels of AT8 phosphorylated tau (ser199/202) within the hippocampus. Consistent with Aβ vaccination studies, sNEP-expressing NSCs have no effect on well-established tau pathology in 21-month-old 3xTg-AD mice (Figure 5A-C). To determine whether sNEP expression leads to a functional effect on neuronal connectivity, we examined synaptophysin immunoreactivity. As shown, sNEP-NSCs increase synaptic density within the subiculum by 31.8% versus control-NSCs (Figure 5D-E, paired t-test, *P* = 0.009). Thus, sNEP-NSCs provide additional benefit by reducing Aβ-mediated synaptotoxicity. Interestingly, synaptic density was not significantly altered within the medial septum or amygdala (data not shown, *P* = 0.41 and 0.54, respectively). This finding could suggest that the distal effects of NEP expression are somewhat diminished. However, synaptic deficits in these two regions have not yet been described in 3xTg-AD mice; thus, changes in the medial septum and amygdala in response to Aβ reduction might not be expected.

To determine the differentiated phenotype of engrafted cells, double-labeling for GFP and neuronal and glial markers was performed. We previously showed that murine NSCs transplanted into aged brains primarily express astrocytic markers [3,20]. In this study we found similar results, detecting very few NSCs that co-expressed the neuronal marker NeuN (Figure 5F), but large numbers of GFP-expressing cells that co-expressed the astrocyte marker GFAP in both sNEP and control-NSC engrafted sides (Figure 5G-L). Similar gliogenic differentiation profiles were observed in both 3xTg-AD (Figure 5) and Thy1-APP mouse models (data not shown).

### Neprilysin-expressing NSCs reduce plaques and increase synaptic density in a second transgenic model of AD

**Figure 6:**
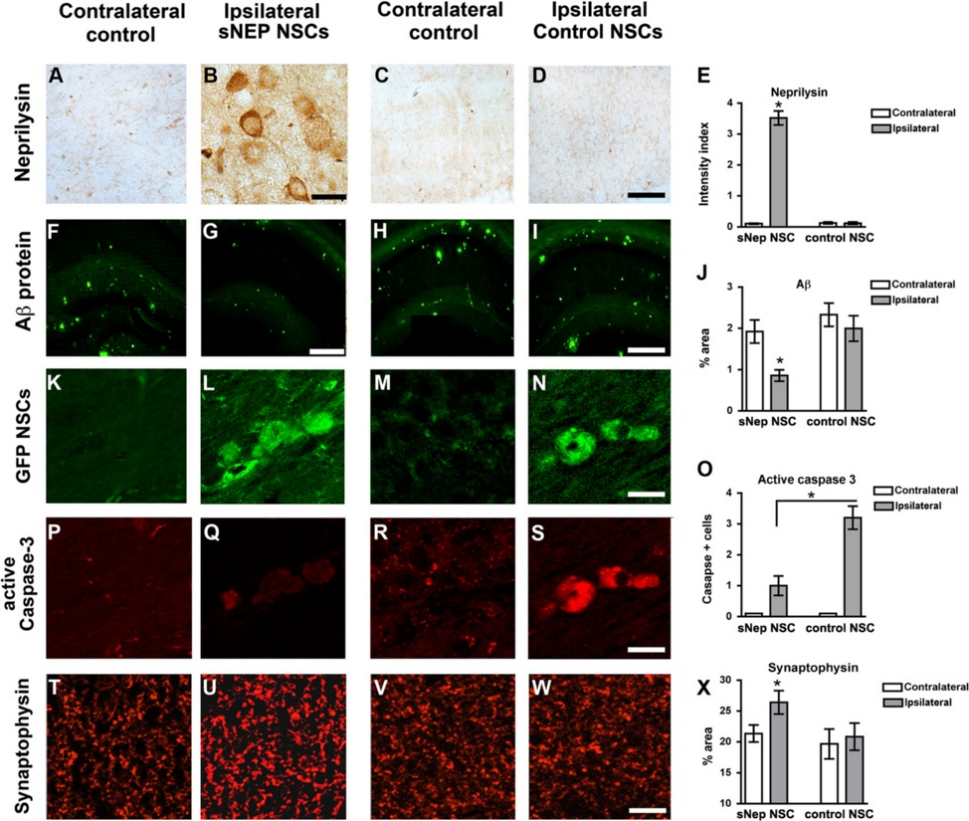
**sNEP-NSCs reduce plaque pathology and resist degeneration in a second transgenic AD model.** Neprilysin immunoreactivity in the contralateral **(A)** and ipsilateral **(B)** hippocampus of sNEP-NSC transplanted transgenic mice reveals high levels of NSC neprilysin expression *in vivo*. **(C-D)** Control NSCs, in contrast, produce little to no neprilysin following transplantation, quantified in **(E)**. At 10 months of age, Thy1-APP mice exhibit considerable amyloidosis (6E10 labelling, green) within the hippocampus **(F)**. However, transplantation of sNEP-NSCs significantly reduced Aβ pathology within the ipsilateral hippocampus **(G)**. Control NSCs by comparison have no effect on Aβ levels **(H-I)**, quantified in **(J)**. GFP labelling (green) reveals examples of NSCs engrafted into the ipsilateral hippocampus **(L,N)**, but not within the contralateral vehicle-injected side of the brain **(K, M)**. In line with *in vitro* findings, caspase activation is reduced by expression of neprilysin **(O)**. Little active caspase-3 immunoreactivity (red) is detected within the ipsilateral hippocampi of transgenic mice **(P, R)**. However, caspase-3 activation (red) within sNEP-NSCs **(Q)** is significantly reduced versus control NSCs **(S)**. Furthermore, levels of the presynaptic terminal marker synaptophysin **(T-X)** are significantly increased by sNEP-NSC transplantation **(U)**, suggesting that neprilysin expression can reduce Aβ-induced synaptotoxicity. N = 6/group, error bars represent standard error of the mean (SEM). Scale Bar = 30 μm in A-D, 350 μm in F-I, 14 μm in K-S, 45 μm in T-W. Aβ, beta**-**amyloid; AD, Alzheimer’s disease; NSCs, neural stem cells; sNEP, secreted neprilysin.

Many transgenic models of AD have been generated and conflicting results have occasionally been observed between different models. We, therefore, tested whether sNEP-expressing NSCs can modify Aβ-levels in a second independent transgenic AD line. For these experiments, sNEP-expressing NSCs were also independently generated and characterized (Figure 2), providing additional evidence regarding the reproducibility of this approach. Thy1-APP mice develop Aβ-plaques beginning at three months of age. To examine the effects of NSCs on established pathology, cells were transplanted into nine-month-old Thy1-APP mice and pathology was examined one month later. As shown, sNEP-expressing NSCs continue to produce NEP one month after transplantation (Figure 6A-E). More importantly, Aβ plaque load is significantly decreased within the ipsilateral hippocampus of sNEP-NSC transplanted mice (Figure 6F-J). In contrast, control-NSCs have no effect on Aβ pathology (Figure 6I,J). Interestingly, sNEP-expression also reduced the activation of caspase-3 within NSCs, suggesting that NEP production likely protects engrafted NSCs from Aβ-induced toxicity (Figure 6K-S). To further examine the potential functional effects of sNEP-expression we examined synaptophysin immunoreactivity in Thy1-APP mice. As in 3xTg-AD mice, we again found that sNEP-NSCs significantly increase synaptic density (Figure 6T-X). Thus, sNEP-expressing NSCs reduce Aβ pathology and improve synaptic connectivity in two independent transgenic models of AD.

### Neprilysin expression may enhance microglial Aβ phagocytosis

**Figure 7:**
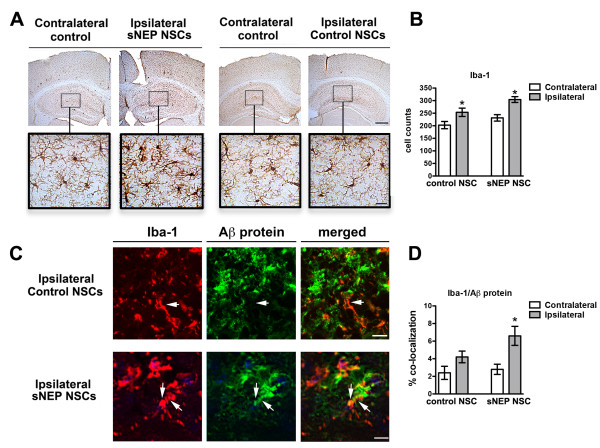
**Neprilysin expression increases microglial Aβ phagocytosis.** The numbers of IBA-1 immunoreactive microglia were quantified adjacent to control and sNEP-NSC grafts. **(A-B)** As expected, NSC transplantation increases the recruitment of IBA-1+ microglia towards the injection site. In addition, sNEP expression appears to further increase graft-associated microglial numbers (*P* <0.05). **(C-D)** Interestingly, neprilysin expression also significantly increases the co-localization between IBA-1+ (red) and Aβ (green), suggesting that sNEP expression may indirectly enhance microglial Aβ phagocytosis (*P* <0.05). N = 6/group, error bars represent standard error of the mean (SEM). Scale Bars = 300 μm in A top panels, 25 μm in A bottom panels, and 12 μm in C. Aβ, beta**-**amyloid; NSCs, neural stem cells; sNEP, secreted neprilysin.

To determine whether NEP expression influences microglial activation state we next examined the microglial marker IBA-1. As one might expect, microglial number was increased adjacent to NSC grafts versus vehicle-injection sites in Thy1-APP mice (Figure 7A,B). Surprisingly, sNEP expression also further elevated microglial number versus control-NSC transplantation. To determine whether this increase in microglial number might further facilitate the degradation of Aβ, we next examined the co-localization of microglia and Aβ. As shown, we found that NSC NEP expression significantly increased the amount of Aβ detected within adjacent microglia (Figure 7C,D). Thus, it appears that NEP expression not only directly degrades Aβ, but may also enhance the microglial degradation of Aβ and/or Aβ fragments in Thy1-APP mice. In contrast to these findings, we found no significant changes in microglial number in response to NEP in 3xTg-AD mice. A likely explanation for this difference is that the Thy1-APP paradigm examined brains after one month duration whereas the 3xTg-AD study investigated the effects of three-months NSC engraftment. It is, therefore, likely that the microglial activation state has significantly diminished by three months post transplantation.

## Discussion

NSC transplantation may provide a promising new approach to treat AD by elevating neurotrophin levels and enhancing endogenous synaptic connectivity [3]. However, NSCs do not modify Aβ pathology; thus, the long-term benefit of NSC transplantation remains unclear. Because of their robust migratory capacity, NSCs provide a compelling approach to deliver therapeutic proteins to the brain. By combining the inherent benefits of NSC transplantation with *ex vivo* gene therapy, modified NSCs may provide a powerful combinatorial approach to treat AD. In this study, we examined whether NEP-expressing NSCs could target and reduce Aβ pathology in two well-established transgenic models of AD. We found that NSCs could be readily modified to express and secrete NEP without altering multipotentcy or differentiation potential. More importantly, sNEP-expressing NSCs dramatically reduced Aβ levels both *in vitro* and *in vivo* and elevated synaptic density in both transgenic models of AD.

We and others previously tested the effect of NEP overexpression in transgenic AD mice [7,10,21,22]. Each of these studies observed significant reductions in Aβ plaque load in response to NEP. We also previously demonstrated improved cognitive function in AD mice following lentiviral delivery of sNEP [21]. In contrast, Meilandt and colleagues found no improvement in cognition when hAPP-J20 mice were crossed to mice overexpressing membrane-bound NEP [22]. Major differences in delivery approach and the use of secreted versus membrane-bound forms of NEP likely account for these differences. In the current study, we again utilized the secreted form of neprilysinNEP, but instead used NSCs as a delivery vehicle. Potential benefits of NSC-mediated delivery over viral methods include a greater distribution of NEP delivery. Whereas NSCs have previously been shown to migrate through the brain parenchema [12], viral-based approaches typically provide only a small radius (approximately 0.5 mm) of infectivity [11]. As the human brain is approximately three thousand times larger than the mouse brain, scale-up of viral gene-therapy for clinical translation remains a considerable challenge. Peripheral delivery of NEP protein also appears to be ineffective, as a recent study found that intravenous delivery of a NEP fusion protein could reduce plasma Aβ, but failed to clear Aβ plaques within the brain [23]. New approaches, such as stem cell mediated delivery, may therefore be needed to provide broader expression of therapeutic proteins in the brain. However, it remains to be determined whether the migratory capacity of NSCs would be sufficient to treat a widespread brain disease such as AD. Clearly NSC-based delivery provides a benefit over current gene therapy approaches, but multiple NSC injections would likely still be needed.

The unilateral transplantation design used in this study allowed us to directly compare plaque load and synaptic density within the same animals. Unfortunately, this design also precluded further biochemical analysis of soluble Aβ. It, therefore, remains possible that soluble Aβ levels are not altered by this approach. However, the observed sNEP-NSC mediated a 31.8% increase in synaptic density, suggesting that soluble Aβ oligomers are also likely reduced. Previous studies also support this notion, as lentiviral-mediated delivery of s-NEP reduces both soluble and insoluble Aβ [21]. As most transgenic AD models, including those used, exhibit little or no neuronal loss we conclude that the effect of sNEP-NSCs on synaptic density is mediated via maintenance and/or enhancement of endogenous synaptic connectivity. It is important to note that synapse loss correlates strongly with cognitive dysfunction in AD patients [24]. Quite notably in the current study we observed a 31.8% increase in hippocampal synaptic density three months after sNEP-NSC transplantation. The magnitude of this effect is similar to the approximately 38% loss of synapses that occurs in AD patients [24]. We, therefore, conclude that the effect of sNEP-NSCs on synaptic density represents a meaningful functional outcome. In addition to Aβ plaques and synaptic loss, cerebral amyloid angiopathy (CAA) represents another important AD-associated pathology. However, neither the 3xTg-AD nor Thy1-APP models develop CAA at the ages studied. Future experiments are therefore needed to determine whether NSC-mediated NEP delivery can also influence CAA.

Other cell types may also be useful for delivering NEP to the AD brain. For example, induced pluripotent stem cells (iPSCs) offer an alternative and extremely promising new cell source that could be used to deliver NEP and personalized cell therapies. Two distinct advantages of iPSC-derived NSCs over allogeneic fetal-derived NSCs include the greatly increased capacity for scale-up and the potential ability to transplant a patient’s own cells, thereby reducing or eliminating the need for immune-suppression. Most recently, xeno-free clinical grade iPSCs have been generated, moving this approach several important steps closer to clinical reality [25].

Peripheral-derived cells are also worth considering. In support of this, Lebson and colleagues transfected CD11b + monocytes with NEP and infused these cells biweekly into AD transgenic mice [26]. Some of these modified monocytes migrated into the brain and Aβ deposition was slowed. This promising approach further supports the notion that cell-based delivery of NEP can be used to target Aβ. However, the use of monocytes offers both advantages and disadvantages in comparison to NSC-based delivery. Monocytes, for example, have limited half-lives; thus, repeated injections are required. This potential drawback can also be viewed as a possible advantage by providing some protection against adverse events. Interestingly, upregulation of NEP, also known as CALLA (common acute lymphoblastic leukemia antigen), commonly occurs in acute leukemias [27]. The overexpression of NEP in hematopoetic lineages is therefore concerning. It is also worth noting that without irradiation, concurrent stroke, or additional treatments, very few monocytes appear to migrate into the AD brain [28]. Thus, monocyte mediated delivery of NEP may not provide as robust a therapeutic approach as NSC-based delivery.

In contrast, our data suggest that the disease-modifying effects of sNEP-NSCs can be extremely robust, modulating Aβ plaques in regions both adjacent to and interconnected with the grafted area. Reductions in local Aβ appear to occur via both NEP-mediated proteolysis and enhanced microglial degradation. The mechanism(s) by which sNEP-NSCs decrease plaque load in more distant regions, such as the amygdala and medial septum, however, remains unclear. Transplanted NSCs did not migrate into the amygdala or medial septum and we were unable to detect clear evidence of axonally-transported NEP. sNEP-NSCs may instead modulate the production and/or anterograde transport of Aβ from the hippocampus to these efferent targets. Intriguingly, recent studies point toward a network diffusion model for the transmission of neurodegenerative pathologies via synaptic networks [29,30]. In light of this, our results could indicate that therapeutic proteins might also effectively modulate pathology via the same neural networks.

## Conclusions

In conclusion, the current study clearly demonstrates that sNEP-expressing NSCs offer a novel and highly effective combinatorial approach to modify AD pathology and enhance synaptic connectivity. Future studies will be needed to determine whether this combinatorial approach can also provide additional long-term behavioral efficacy and whether such approaches can be translated towards an eventual clinical application.

## Abbreviations

3xTg-AD: triple transgenic mouse model of Alzheimer’s Disease; AD: Alzheimer’s Disease; ANOVA: analysis of variance; BDNF: brain-derived neurotrophic factor; Aβ: beta-amyloid; CALLA: common acute lymphoblastic leukemia antigen; (D)MEM: (Dulbecco's) modified Eagle’s medium; EGF: epidermal growth factor; ELISA: enzyme-linked immunosorbent assay; GFAP: glial fibrillary acid protein; GFP: green fluorescent protein; LDH: lactate dehydrogenase; MHC: major histocompatibility complex; NEP: neprilysin; NFTs: neurofibrillary tangles; NSC: neural stem cell; PBS: phosphate-buffered saline; PLSD: protected least significant difference; ROI: region of interest; sNEP: secreted neprilysin; sox2: sex determining region Y-box 2; Thy1: thymus cell antigen 1.

## Competing interests

The authors declare they have no competing interests.

## Authors’ contributions

MBJ: conception and design, 3xTg-AD transplantation studies, data collection and analysis, financial support, manuscript writing and final approval of the manuscript. BS: generation of neprilysin constructs and viruses, data collection and analysis, final approval of the manuscript. SM: Thy1-APP transplantation studies, data collection, and analysis, final approval of the manuscript. NAC: 3xTg-AD transplantation studies, data collection and analysis, final approval of the manuscript. AAA: 3xTg-AD transplantation studies, data collection and analysis, and final approval of the manuscript. JLD: 3xTg-AD transplantation studies, data collection and analysis, and final approval of the manuscript. FJM: conception and design, manuscript writing and final approval of the manuscript. JFL: conception and design, manuscript writing and final approval of the manuscript. EM: conception and design, data collection and analysis, financial support, manuscript writing and final approval of the manuscript. FML: manuscript writing, financial support, and final approval of the manuscript. All authors read and approved the final manuscript.
