# Correction: Neural stem cells genetically-modified to express neprilysin reduce pathology in Alzheimer transgenic models

**DOI:** 10.1186/s13287-024-03702-7

**Published:** 2024-03-25

**Authors:** Mathew Blurton-Jones, Brian Spencer, Sara Michael, Nicholas A. Castello, Andranik A. Agazaryan, Joy L. Davis, Franz-Josef Müller, Jeanne F. Loring, Eliezer Masliah, Frank M. LaFerla

**Affiliations:** 1 Department of Neurobiology and Behavior and Institute for Memory Impairment and Neurological Disorders, University of California Irvine, Irvine, CA 92697 USA; 2 Center for Regenerative Medicine, the Scripps Research Institute, La Jolla, CA 92037 USA; 3 Center for Psychiatry (ZIP Kiel), University Hospital Schleswig Holstein, 24105 Kiel, Germany; 4 Department of Neurosciences, University of California San Diego, La Jolla, CA 92093 USA

**Correction: Stem Cell Research & Therapy 2014, 5:46** 10.1186/scrt440

The original article contains an artifact in Fig. 6H that obscures the view of the lower-middle portion of the image. The correct original image for Fig. 6H can be viewed in this Correction article. 

**Fig. 6:**
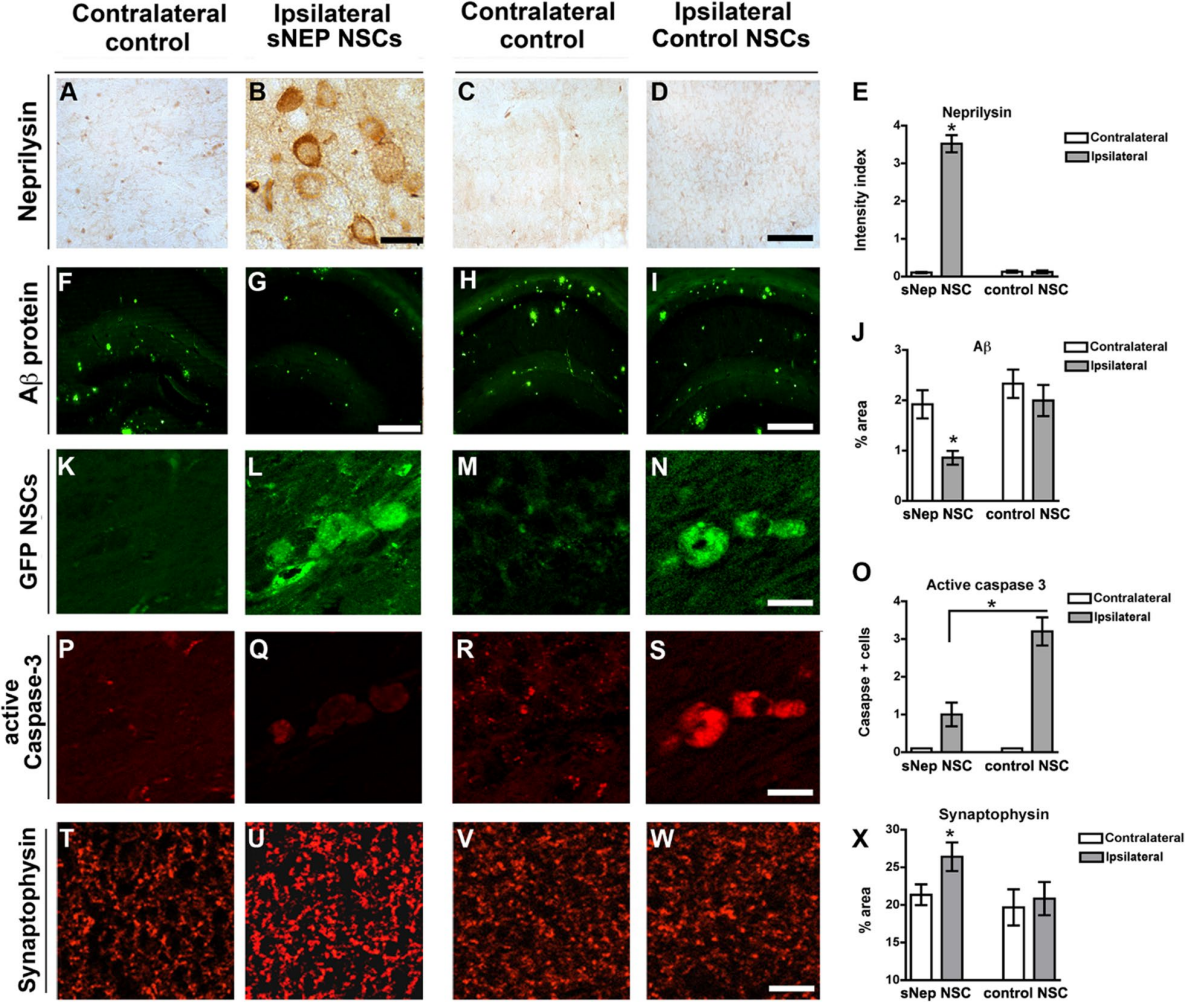
sNEP-NSCs reduce plaque pathology and resist degeneration in a second transgenic AD model. Neprilysin immunoreactivity in the contralateral (**A**) and ipsilateral (**B**) hippocampus of sNEP-NSC transplanted transgenic mice reveals high levels of NSC neprilysin expression in vivo. (**C**-**D**) Control NSCs, in contrast, produce little to no neprilysin following transplantation, quantified in (**E**). At 10 months of age, Thy1-APP mice exhibit considerable amyloidosis (6E10 labelling, green) within the hippocampus (**F**). However, transplantation of sNEP-NSCs significantly reduced Aβ pathology within the ipsilateral hippocampus (**G**). Control NSCs by comparison have no effect on Aβ levels (**H**-**I**), quantified in (**J**). GFP labelling (green) reveals examples of NSCs engrafted into the ipsilateral hippocampus (**L**, **N**), but not within the contralateral vehicle-injected side of the brain (**K**, **M**). In line with in vitro findings, caspase activation is reduced by expression of neprilysin (**O**). Little active caspase-3 immunoreactivity (red) is detected within the ipsilateral hippocampi of transgenic mice (**P**, **R**). However, caspase-3 activation (red) within sNEP-NSCs (**Q**) is significantly reduced versus control NSCs (**S**). Furthermore, levels of the presynaptic terminal marker synaptophysin (**T**-**X**) are significantly increased by sNEP-NSC transplantation (**U**), suggesting that neprilysin expression can reduce Aβ-induced synaptotoxicity. N = 6/group, error bars represent standard error of the mean (SEM). Scale Bar = 30 μm in A-D, 350 μm in F-I, 14 μm in K-S, 45 μm in T-W. Aβ, beta-amyloid; AD, Alzheimer’s disease; NSCs, neural stem cells; sNEP, secreted neprilysin.

